# A statistical method to identify recombination in bacterial genomes based on SNP incompatibility

**DOI:** 10.1186/s12859-018-2456-z

**Published:** 2018-11-22

**Authors:** Yi-Pin Lai, Thomas R. Ioerger

**Affiliations:** Department of Computer Science & Engineering, Texas A&M University, College Station, TX 77843 USA

**Keywords:** Recombination, Compatibility, Bacteria, Evolution, Phylogenetics

## Abstract

**Background:**

Phylogeny estimation for bacteria is likely to reflect their true evolutionary histories only if they are highly clonal. However, recombination events could occur during evolution for some species. The reconstruction of phylogenetic trees from an alignment without considering recombination could be misleading, since the relationships among strains in some parts of the genome might be different than in others. Using a single, global tree can create the appearance of homoplasy in recombined regions. Hence, the identification of recombination breakpoints is essential to better understand the evolutionary relationships of isolates among a bacterial population.

**Results:**

Previously, we have developed a method (called ACR) to detect potential breakpoints in an alignment by evaluating compatibility of polymorphic sites in a sliding window. To assess the statistical significance of candidate breakpoints, we propose an extension of the algorithm (ptACR) that applies a permutation test to generate a null distribution for comparing the average local compatibility. The performance of ptACR is evaluated on both simulated and empirical datasets. ptACR is shown to have similar sensitivity (true positive rate) but a lower false positive rate and higher F1 score compared to basic ACR. When used to analyze a collection of clinical isolates of *Staphylococcus aureus*, ptACR finds clear evidence of recombination events in this bacterial pathogen, and is able to identify statistically significant boundaries of chromosomal regions with distinct phylogenies.

**Conclusions:**

ptACR is an accurate and efficient method for identifying genomic regions affected by recombination in bacterial genomes.

## Background

Recombination is an important force of evolution in prokaryotes that results in genetic exchange, usually involving transformation, transduction and conjugation [1]. In bacterial populations, when some strains acquire genetic changes from other strains, it can produce the appearance of homoplasy (where the same change at a site appears to have occurred multiple times independently, in separate branches). In a multiple sequence alignment, the polymorphic sites may have different phylogenetic relationships compared with other sites, i.e., phylogenetic incongruence [2, 3]. Studies have explored the effect of recombination in phylogeny estimation and indicated that the impact depends on the extent of recombinant events and the relatedness of taxa [1, 4, 5]. The true evolutionary history of a set of taxa may not be reflected if recombination events occurred during evolution yet are ignored. Growing evidence indicates that recombination has occurred in the evolution of many pathogenic bacterial species, including *Mycobacterium avium* [6], *Mycobacterium intracellulare* [7], *Neisseria meningitidis* [8, 9], *Salmonella enterica* [10], *Staphylococcus aureus* [11–13], *Streptococcus pneumoniae* [14] and *Streptococcus pyogenes* [15]. Hence, it is essential to identify recombination regions among bacterial isolates before inferring a phylogeny, to better understand their evolutionary histories.

Over the last four decades, many methods have been proposed to detect the presence of recombination in bacterial genomes, applying concepts of maximum likelihood, phylogenetic incongruence, substitution patterns, distance-based approach, or character compatibility [16–22]. Commonly used methods to identify recombination breakpoints include ClonalFrameML [22], RDP [18] and GARD [19]. All are phylogenetic-based programs. ClonalFrameML utilizes a maximum-likelihood tree to reconstruct ancestral states of internal nodes. It then applies a hidden Markov model (ClonalFrame) to infer the recombination parameters and recombination locations of each branch of the tree using an Expectation-Maximization (EM) algorithm [22]. RDP characterizes homoplasy signals using pairwise scanning of the alignment, with the integration of several non-parametric recombination detection methods [18]. GARD applies Akaike’s Information Criterion with a genetic algorithm to search the recombinant locations heuristically [19]. Compatibility-based methods are considered to be more efficient than phylogenetic-based methods to identify recombination, since they do not require the reconstruction of phylogenetic trees [16]. The Reticulate program uses compatibility matrices to calculate a neighbor similarity score (NSS), and clusters compatible sites by randomly shuffling the matrices [17]. Bruen et al. define the pairwise homoplasy index (PHI) in terms of a pairwise incompatibility score of each site and its downstream sites in a global alignment, and then they obtain a *p*-value by computing the cumulative probability under a normal distribution generated from expected mean and variance of the PHI statistic [20]. Both programs are compatibility-based methods and able to detect recombination and report informative sites, but they do not report breakpoints.

(paragraph on compatibility integrated into Methods...)

In our previous work, an average compatibility ratio (ACR) method was introduced to identify the potential recombination breakpoints in a bacterial genome by analyzing the pattern of SNPs among a collection of isolates using a sliding window [23]. The ACR method detects the presence or absence of recombination by calculating an overall compatibility score among pairs of sites. Next, ACR will scan the entire alignment with a sliding window of fixed size to identify regions where the local compatibility among pairs of sites in the region decreases and reaches a local minimum. However, the local minima may include false positives. In this paper, we propose the use of a permutation test on the positions of local minima to assess the statistical significance of potential breakpoints in the genome. We also extend the ACR method to test the compatibility of multi-state characters by applying an efficient algorithm based on Buneman’s theorem [24]. The performance of ptACR is evaluated on simulated datasets with varying mutation rates and rate heterogeneity among sites. The sequences are simulated by evolving along distinct trees with changes in topology, where a group of taxa have been moved from one branch to another randomly. The simulation results show that the integration of the permutation test has lower false positive rate than basic ACR method. Yet both methods have a similar level of sensitivity for the detection of recombination breakpoints. We use ptACR to identify genomic regions of recombination in clinical isolates of *Staphylococcus aureus*.

## Methods

### Characters and compatibility

The concept of compatibility was initially described by LeQuesne in 1969 for binary-state characters [25]. For a multiple DNA sequence alignment, a character is defined as a set of states (nucleotides) for all taxa at a given site. A binary character is a polymorphic site with 2 nucleotides. Two binary-state characters are compatible if a single phylogenetic topology is enough to explain both characters:

#### **Definition 1**

Pairwise compatibility for binary characters: Two sites of binary characters are compatible if and only if there exists a tree for which each site can be explained by one change.

For a pair of binary characters at two sites, the four gamete test is a quick way in polynomial time to determine their compatibility [26]. It converts the state of taxa at each site to 0 and 1, and concatenates the states at two sites for a given taxon as one of the following combinations: {00, 01, 10, 11}. If at most three combinations exist, then the two sites are compatible. For a set of binary characters in an alignment, there exists a perfect phylogeny if all characters are jointly compatible. For a set of 3 or more binary-state sites in a region of genome, if all pairs of sites are pairwise compatible, then they are jointly compatible, i.e. a tree exists that can explain all sites.

More generally, in a whole-genome alignment of multiple taxa, some sites can also have multiple states, e.g., 3 or 4 nucleotides.

#### **Definition 2**

Pairwise compatibility for multi-state characters: Two sites of multi-state characters are compatible if and only if there exists a tree for which each site can be explained by number of change that equals to number of distinct states minus one (the minimum number of changes required for a site with *n* nucleotides is *n*-1).

To determine the compatibility of a pair of multi-state characters (two sites at a time), the problem can be reduced to triangulating colored graphs problem [27] and then solved in polynomial time [24]. Two characters are firstly converted to a partition intersection graph by the following steps. For each character, the taxa of the same state are denoted as a vertex. An edge between two vertices is added if the vertices contain the same taxon/taxa to form the partition intersection graph. Next, if their derived partition intersection graph is acyclic, then they are determined to be compatible [24]. The method to determine the compatibility of two characters is illustrated in Algorithm 1. For multi-state characters, pairwise compatibility does not guarantee setwise compatibility.The question of determining whether a set of *n*>2 multi-state sites is compatible is reducible to the problem of finding the maximum clique, which is NP-complete [24].


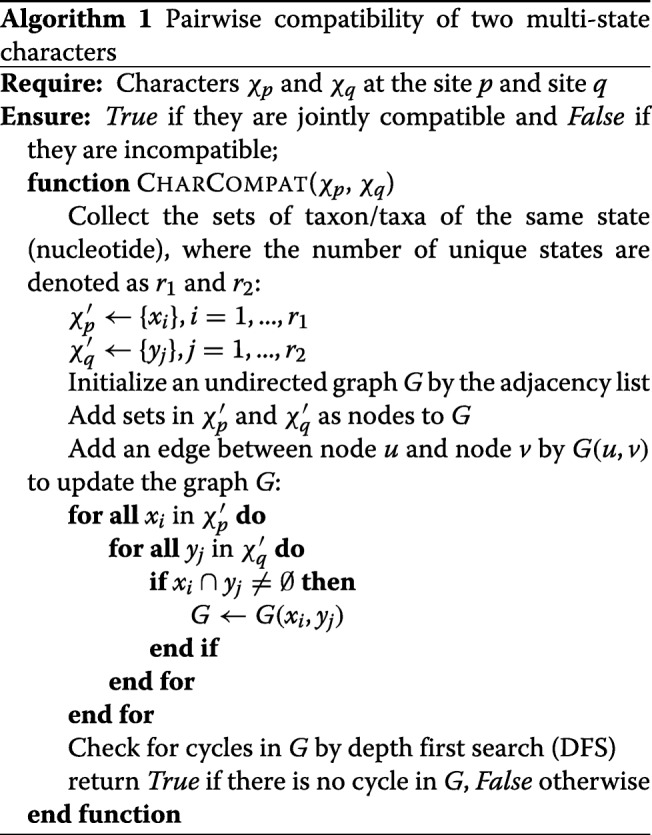


Given a multiple sequence alignment of *n* taxa and *m* informative sites, at each informative site *i*, ACR calculates a pairwise compatibility score between all pairs of informative sites within a sliding window of size 2*w* centered on the *i*^*th*^ SNP (from *i*-*w* to *i*+*w*). The pairwise compatibility score is 1 if characters *χ*_*p*_ and *χ*_*q*_ are compatible; otherwise, the score is 0 (Eq. 1). Next, it averages the scores of all pairs of sites within the region to obtain the average compatibility ratio, $\sigma _{i_{w}}$, for the region (Eq. 2).

1 $$ {CompatPW}_{pq} \,=\, \!\left\{\begin{array}{ll} \!1, & \!\text{if characters }\chi_{p} \text{ and } \chi_{q} \text{ are compatible}\\ \! 0, & \!\text{otherwise} \end{array}\right.   $$

2 $$ \sigma_{i_{w}} = \frac{1}{\left({2w+1\atop2}\right)}\sum\limits_{p=i-w}^{i+w-1} \sum\limits_{q=p+1}^{i+w} {CompatPW}_{pq}   $$

**Fig. 1:**
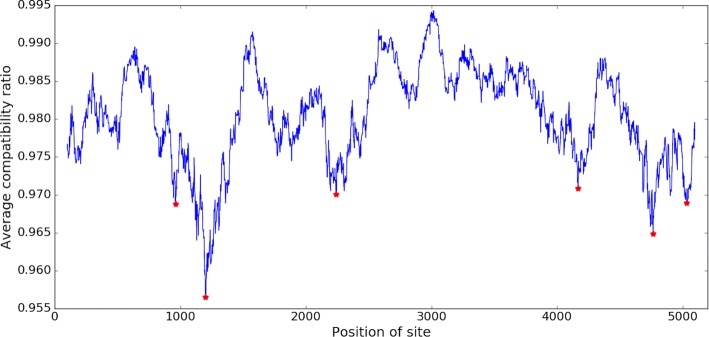
Example of applying ACR on an alignment of several recombined regions using the window size of 200. Among 5200 sites, six sites are identified as the potential breakpoints and labeled in red

The lower the value of the average compatibility ratio ($\sigma _{i_{w}}$), the less jointly compatible the sites in a window are. Hence, a site of local minimum means that sites in the region are least compatible locally, suggesting phylogenetic incongruence between the upstream and downstream regions. Sites with local minima of average compatibility ratio are regarded as potential breakpoints. An example of applying ACR on a recombined alignment of 5200 sites using the window size of 200 is demonstrated in Fig. 1.

To assess the statistical significances of potential breakpoints, we apply a permutation test. The test statistic, $s_{i_{w}}$, for a potential breakpoint at the site *i* is defined as the summation of all compatibility scores of pairs composed of a site from the upstream region [*i*−*w*,*i*−1] with the other site from the downstream region [*i*+1,*i*+*w*] (Eq. 3).

3 $$ s_{i_{w}} = \sum\limits_{p=i-w}^{i-1} \sum\limits_{q=i+1}^{i+w} {CompatPW}_{pq}   $$

This statistic is compared to a null distribution generated by permuting the sites in the window. The null hypothesis is that the level of compatibility between the sites in the window is independent of the sequential order of the sites, i.e. whether sites are compared from upstream or downstream of site *i* does not matter. The alternative hypothesis is that the order of the sites in the local sequences is crucial and does not happen by chance. So the sites within the region are randomly shuffled multiple times (default: 10,000) to produce the sampling distribution of values $s_{i_{w}}$ obtained under the null hypothesis. Let the distribution of values from random permutations on sites in the window be denoted by *D*_*s*_. The significance of observed value $s_{i_{w}}$ is determined by computing the proportion of times that the permuted statistics in *D*_*s*_ are less than or equal to the observed value to get the empirical *p*-value (Eq. 4).

4 $$ p = P\left(x \leq s{_{i_{w}}} \text{ for} x \in D_{s}\right)   $$

**Fig. 2:**
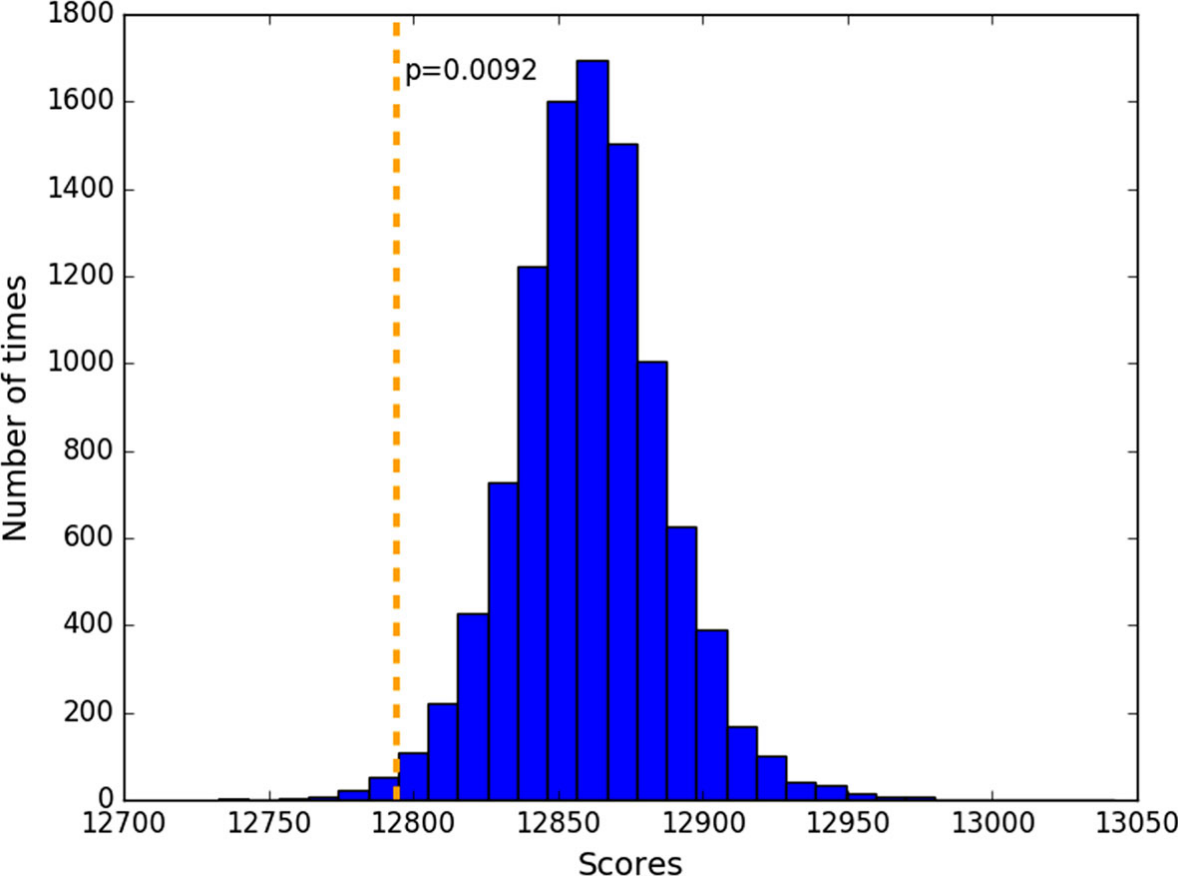
Example of the assessment of statistical significance for a compatibility score in the histogram of a null distribution (N=10k). Observed compatibility score at the site *i* was 12800, among pairs selected upstream and downstream sites. Distribution shows scores from randomly selected pairs in window of [ *i*−*w*, *i*+*w*]. The *p*-value in this case is 0.0092 (at the tail)

If the *p*-value is lower than a given threshold (default: 0.05), then it rejects the null hypothesis of no recombination, hence ptACR will report the site as a probable/significant breakpoint. To correct the *p*-value threshold due to multiple comparison, we use the Bonferroni correction and set the adjusted *p*-value cutoff to 0.05/*n*, where *n* is the number of local minima identified by ACR, to limit the false discovery rate to at most 5%. An example of a statistic determined as significant in the histogram of a null distribution is illustrated in Fig. 2. To make the permutation test more efficient, we convert all characters in nucleotides of the alignment to patterns in numbers and make character patterns as a unique set. Then we record pairwise compatibility information among all pairwise patterns in the set in a hash table. Hence, the compatibility information of any two shuffled sites can be looked up in the hash table in constant time.

### Estimation of phylogenies and homoplasy

Given a sorted list of candidate breakpoints, local phylogenetic trees of each region between two adjacent breakpoints is constructed by the maximum parsimony method using the function of *dnapars* in PHYLIP 3.66 [28]. To estimate the level of homoplasy for each region, the homoplasy ratio and excess changes are calculated by applying the Sankoff Algorithm [29] on each local tree. The *homoplasy ratio*, which is also called the ratio of changes per site, is defined as the summation of actual state changes (Sankoff score) divided by the summation of minimum number of changes (number of nucleotides at each site minus one). The number of *excess changes* for a site is defined as the difference between the number of actual changes and minimum number of changes. For a given region, the homoplasy ratio of 1.0 means all sites are congruent (homoplasy-free); a homoplasy ratio >1.0 means some sites are homoplasic, requiring excess changes in the maximum-parsimony tree.

### Performance on simulated datasets

To evaluate the performance of ptACR, we generated simulated sequence data with known recombinations by random branch swaps. Our goal was to evaluate the sensitivity and specificity of detecting known breakpoints, and how this depends on mutation rate and differences in topology. To simulate sequences with predetermined recombination events, a bifurcating tree with 10 taxa is generated by GenPhyloData [30] under a birth-death process with a birth rate of 0.2 and a death rate of 0.1. Next, 300 alternative trees with recombination between a random pair of donor and acceptor branches based on the original tree are obtained using HGT-Gen [31]. Then, Seq-Gen 1.3.4 [32] is applied to generate aligned sequences of 1000 sites evolved along each tree. Parameters for substitution rate and heterogeneity are varied in the experiment, as described below. The sequences are simulated under the Hasegawa-Kishino-Yano model (HKY85) [33] with nucleotide frequencies A:0.2, G:0.3, C:0.3, T:0.2 and 2-to-1 ratio of transitions to transversions. Lastly, we concatenate sequences for the original tree, one of the modified trees, and the original tree again to obtain a simulated alignment with 3000 total sites that has recombination breakpoints around coordinates 1000 and 2000 and a distinct phylogeny in the middle.

The true positive rate (*sensitivity*), false positive rate (1-*specificity*), and F1 score for the ptACR method are defined as follows. For an alignment with a predetermined recombination region, the inferred breakpoint that is located within 50 bp of an actual breakpoint (ground truth) is counted as true positive (TP), and one that is identified by our method but not within this range is denoted as false positive (FP). Failure to detect a known breakpoint at any site within 50 bp is counted as false negative (FN). The true and false positive rates are defined by dividing by the total number of true breakpoints, and the total number of negative sites outside the breakpoint windows, respectively, $\frac {TP}{TP+FN}$ and $\frac {FP}{FP+TN}$. The precision is defined as the number of accurately inferred breakpoints to the number of identified breakpoints, $\frac {TP}{TP+FP}$. The F1 score, which is the harmonic mean of sensitivity and precision, is $\frac {TP}{2TP+FP+FN}$; higher F1 is better. For each scenario, we average the statistics over all the replicates.

#### Effect of evolutionary distance

Because recombination events among deeper branches should involve strains with more differences and make incompatibility easier to detect, we expect that sensitivity and specificity will vary as a function of the magnitude of the changes in the simulated trees. To quantify this, we defined an metric called evolutionary branch swapping distance (EBSD) to divide the alternative trees into 3 groups: small, medium, and large evolutionary changes. While there are several generalized methods for comparing topologies of arbitrary labeled trees (sharing the same taxa) [34–36], assuming that the change between two trees involves only a single branch swap (as generated by HGT-Gen, simulating recombination), we developed a quantitative measure that reflects the magnitude of evolutionary distance involved in the change. First, we identify the group of taxa that changes position in the tree. Call this group A, and let B be the complement in the tree (rest of the taxa). We define the evolutionary branch swapping distance between the two trees (T1 and T2) as the average absolute value of the difference in distances between each pair of taxa *i* in A and *j* in B in trees T1 and T2 (Eq. 5).

5 $$ EBSD(T1,T2) =\! \frac{1}{|A|*|B|} \sum\limits_{i \in A} \sum\limits_{j \in B} | {dist}_{T1}(i,j)-{dist}_{T2}(i,j)|   $$

**Fig. 3:**
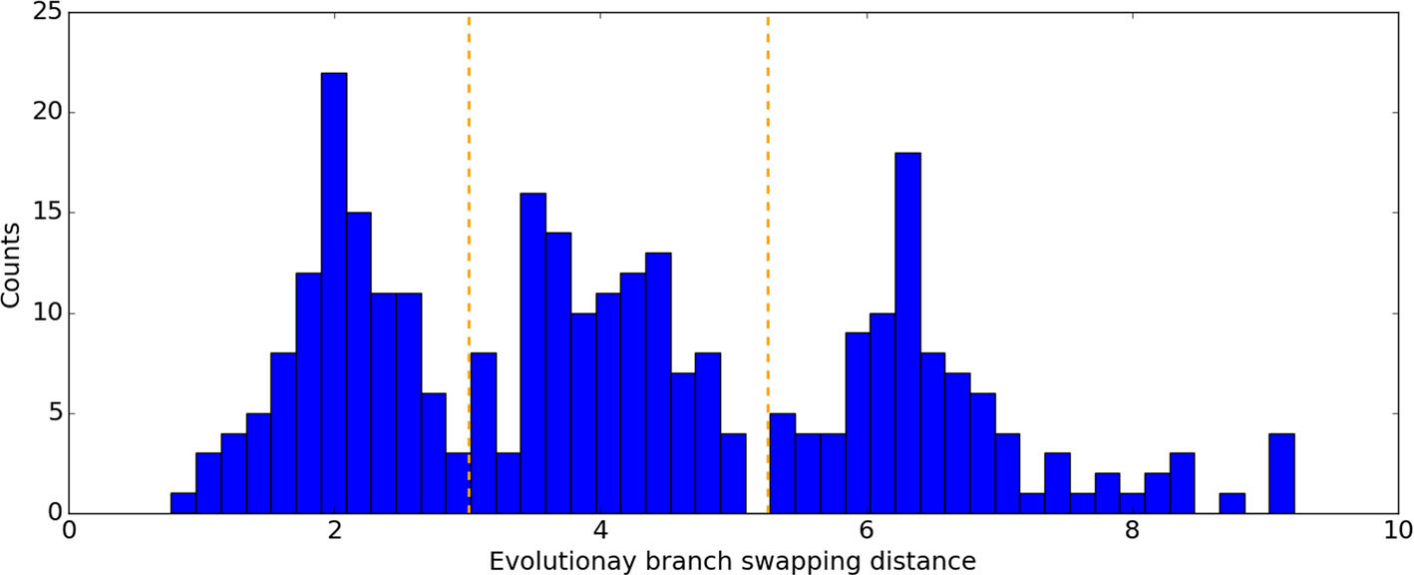
Histogram of evolutionary branch swapping distance between the original tree and 300 alternative trees generated using HGT-Gen

The distance between two taxa is defined as the sum of branch lengths on the connecting path in a tree. The distances between pairs of taxa that are both in A or both in B should be unaffected by the branch swap; only pairs of strains between the two groups will exhibit changes in relative position, and hence changes in distance. If a strain (or group of strains) recombines with a nearby branch, the average change of distances among them will be small; however, if they recombine with a more remote branch of the tree, representing exchange of genetic material with a more divergent strain, then the change in relationships will be more pronounced, and the average change in relative distances among the strains will be larger. The distribution of EBSD distances between the original tree and the 300 alternative trees ranged from 0.77 to 9.22 (see histogram in Fig. 3). The alternative trees are categorized into three groups according to the tree distance with the original one, including small (<3.0), medium (3.0-5.0) and large distance (>5.0) groups. There are about 100 trees in each category.

**Fig. 4:**
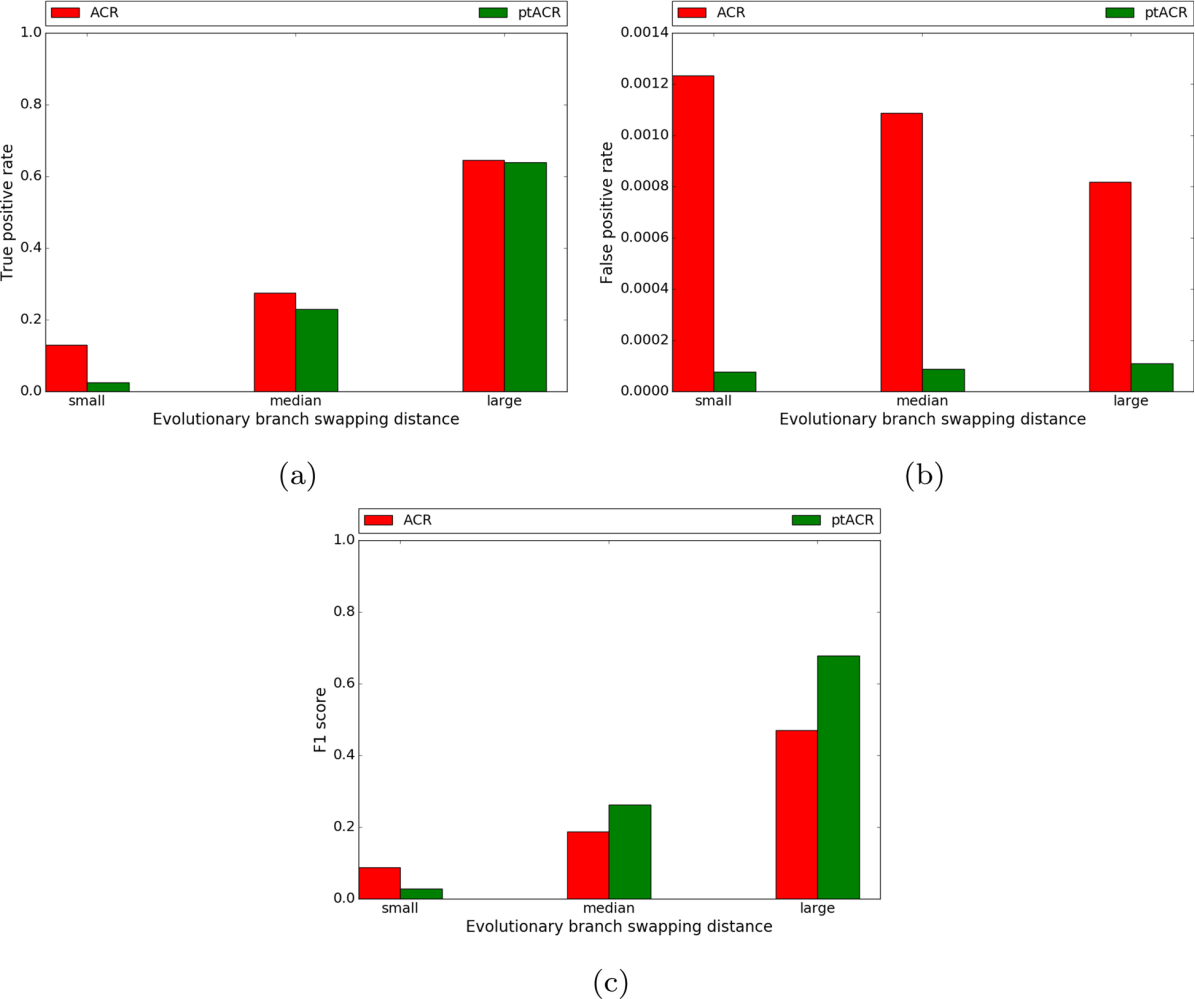
True positive rate (**a**), false positive rate (**b**) and F1 score (**c**) of 3 scenarios of increasing evolutionary branch swapping distance (no heterogeneity)

The true positive rate, false positive rate and F1 score of replicates in the three groups are shown in Fig. 4. Importantly, there is a great reduction in false positives (Fig. 4b) without much loss of true positives (Fig. 4a) for ptACR on ACR. In general, a replicate in the large evolutionary branch swapping distance group has sequences simulated from a more distinct alternative topology compared to the original tree, which makes the sites in the middle of the alignment tend to exhibit more homoplasy. Thus, the boundaries of the recombination event are easier to detect. In contrast, replicates in the small distance group have closer relatedness of taxa since the alternative tree is less different to the original tree. As evolutionary branch swapping distance decreases, both sensitivity and specificity are reduced.

#### Effect of substitution rate and heterogeneity

**Fig. 5:**
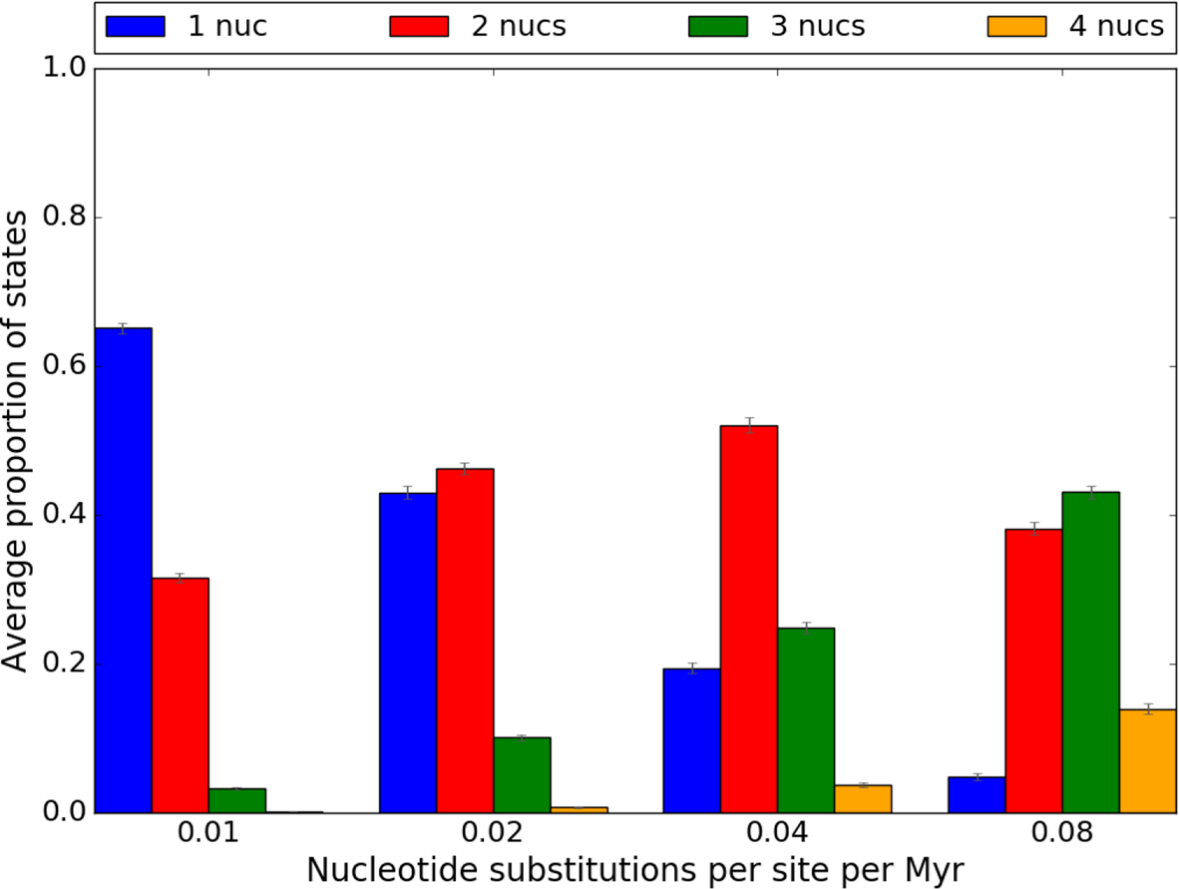
Proportion of nucleotides in 4 scenarios of increasing substitution rate

**Fig. 6:**
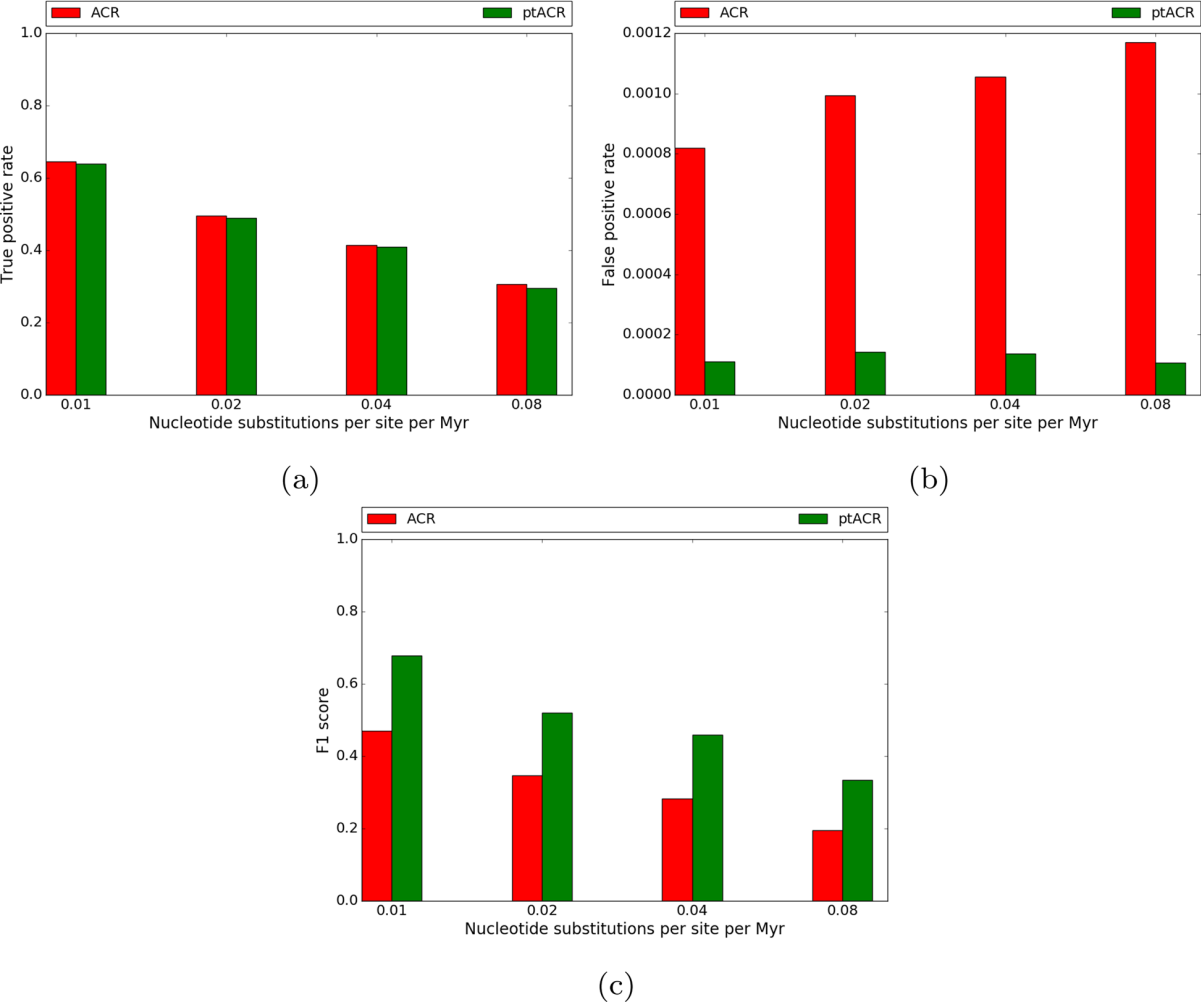
True positive rate (**a**), false positive rate (**b**) and F1 score (**c**) of 4 scenarios of increasing substitution rate

Sequences were simulated in four scenarios by setting the substitution rate parameter of Seq-Gen to 0.01, 0.02, 0.04 and 0.08. Only recombined trees in the large evolutionary branch-swap distance group were used in this experiment, as the sensitivity of ptACR is higher. The default substitution rate heterogeneity parameter in Seq-Gen was used (*α*=*∞*, which means no heterogeneity). The proportion of nucleotides in each scenario is shown in Fig. 5. With low substitution rate, there are 62% monomorphic sites. As substitution rate increases, the fraction of informative sites increases. The true positive rate, false positive rate and F1 score of the four scenarios are plotted in Fig. 6. With low substitution rate, the true positive rate is high, the false positive rate is low and the F1 score is high. The ptACR approach performs better than the ACR in terms of lower false positive rate and higher F1 score.

**Fig. 7:**
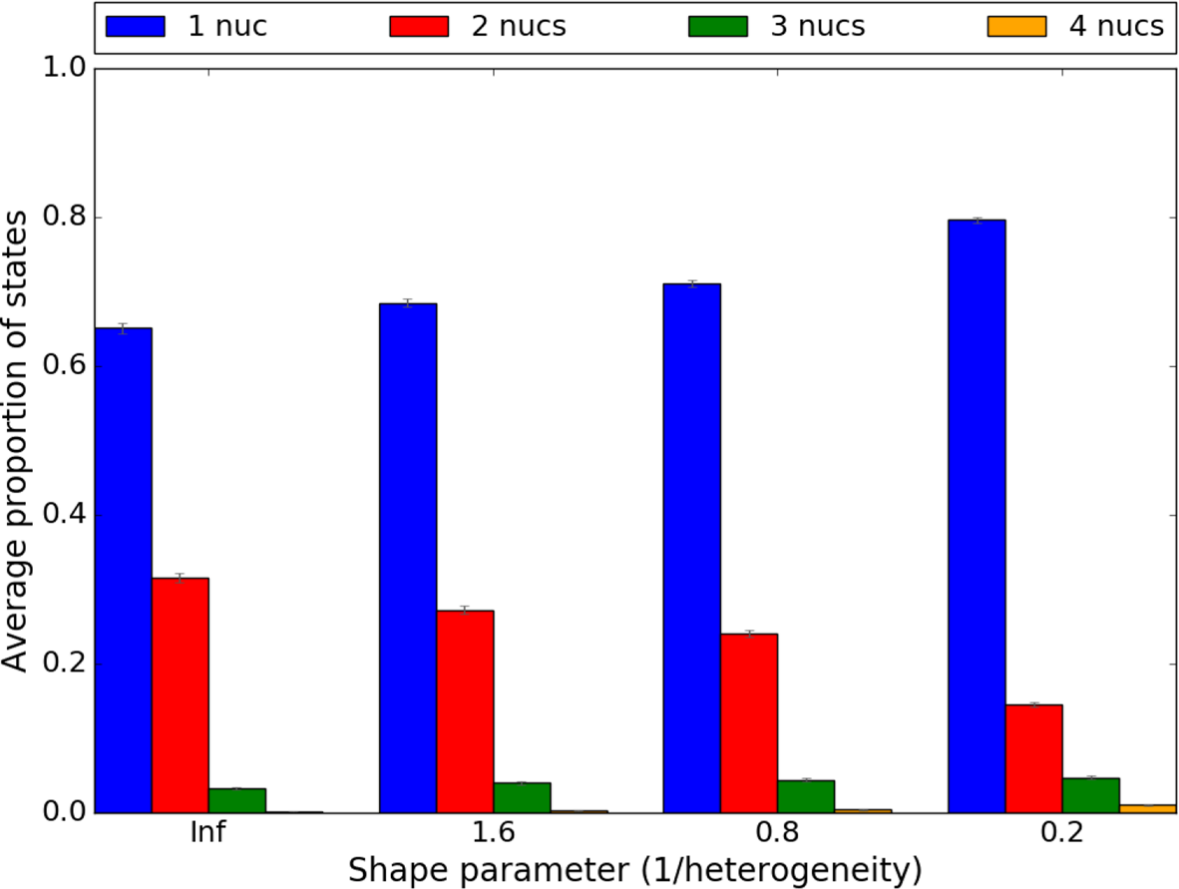
Proportion of nucleotides in 4 scenarios of increasing heterogeneity

**Fig. 8:**
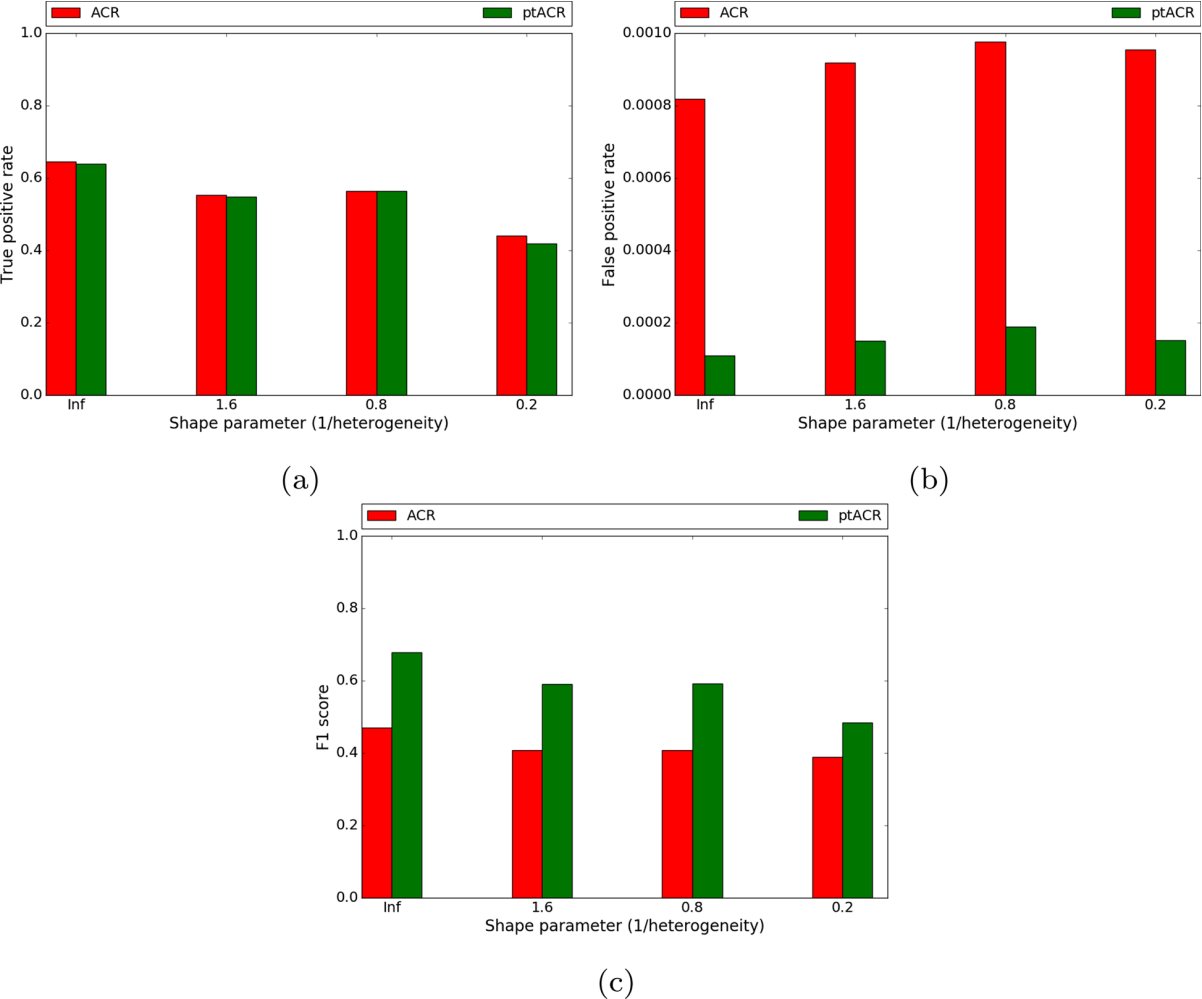
True positive rate (**a**), false positive rate (**b**) and F1 score (**c**) of 4 scenarios of increasing heterogeneity (fixed substitution rate)

To examine how substitution rate heterogeneity affects ptACR performance, we varied the heterogeneity *α* (shape parameter of the gamma distribution) in Seq-Gen, which influences the variability of substitution rates among individual sites. Sequences are simulated in four scenarios of heterogeneity parameter *α* ranging from 0.2, 0.8, 1.6 to *∞* (with the fixed substitution rate of 0.01). The scenario where *α* is equal to *∞* represents sequences simulated with a uniform rate at all sites. The proportion of nucleotides in alignments in each scenario is listed in Fig. 7. With low heterogeneity (*α*=*∞*), there are 37% polymorphic sites and 12% of there are multi-state characters. As heterogeneity increases, the fraction of informative sites decreases. The true positive rate, false positive rate and F1 score of four scenarios are plotted in Fig. 8. The red bars stand for the results from the previous ACR method while the green bars show the results of incorporating the permutation test (ptACR). With low heterogeneity, the true positive rate is high, the false positive rate is low and the F1 score is high. Only at the highest heterogeneity are the sensitivity and specificity reduced. Hence, ptACR accurately detects recombination breakpoints in the alignments, including multi-state characters, except in the most extreme divergent situations (where there is more background homoplasy) occurring stochastically even without recombination.

## Results

**Fig. 9:**
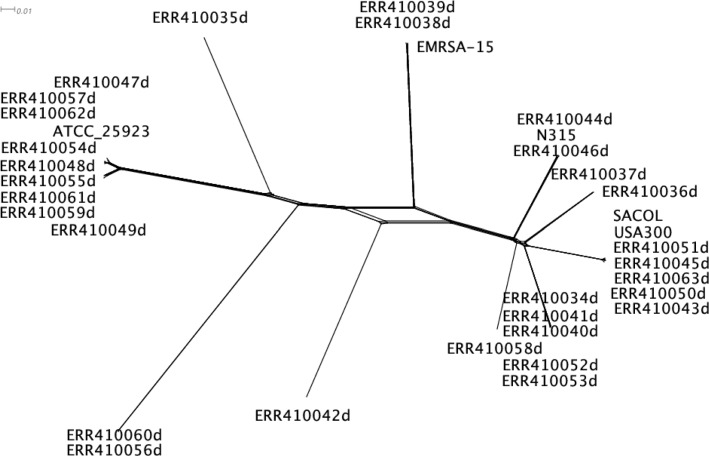
Global phylogenetic tree of 35 strains for *S. aureus*. This figure was produced using SplitsTree [37]. The network of parallel edges indicates that sites exist that are not congruent with a perfect monophyletic tree

We applied ptACR to analyze a collection of 30 clinical isolates of *Staphylococcus aureus* [12] aligned with 5 reference strains, including ST8:USA300 (NC_010079.1), SACOL (CP000046.1), EMRSA-15 (HE681097.1), N315 (BA000018.3) and ATCC 25923 (NZ_CP009361.1). Recombination has previously been observed for the species [12, 13]. The alignment of *Staphylococcus aureus* contains 2.87 Mb nucleotides where 113,936 sites are informative (polymorphic) and 3,625 sites (3.18%) have over two nucleotides. The overall compatibility ratio over the genome is 88.34% and the homoplasy ratio is 1.4484, suggesting recombination occurs among the population. The global phylogenetic tree is shown in Fig. 9.

**Fig. 10:**
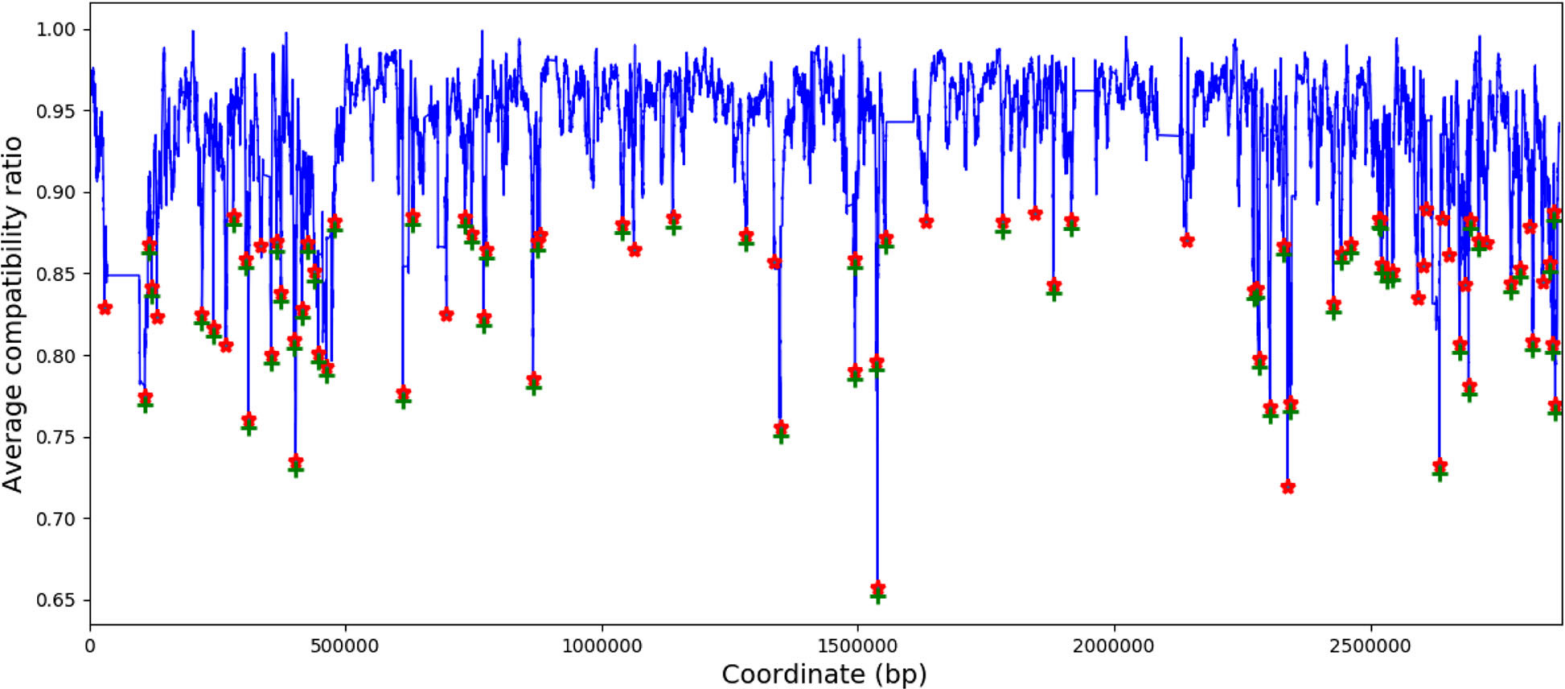
Identified breakpoints using window sizes of 250 informative sites for *S. aureus*

Figure 10 illustrates that 86 local minima (labeled in red) are identified by ACR as potential breakpoints using a window size of 250 informative sites, and then 65 breakpoints (labeled in green) are identified as statistically significant by ptACR with permutation test, where the Bonferroni-adjusted *p*-value threshold is 0.000581 (0.05/86). Hence, 66 regions are obtained.

Any two adjacent regional phylogenetic trees constructed by their corresponding local alignments have distinct tree topologies, reflecting the identified boundaries are confident, since changes in phylogenetic relationships occur between each pair of adjacent regions.

**Fig. 11:**
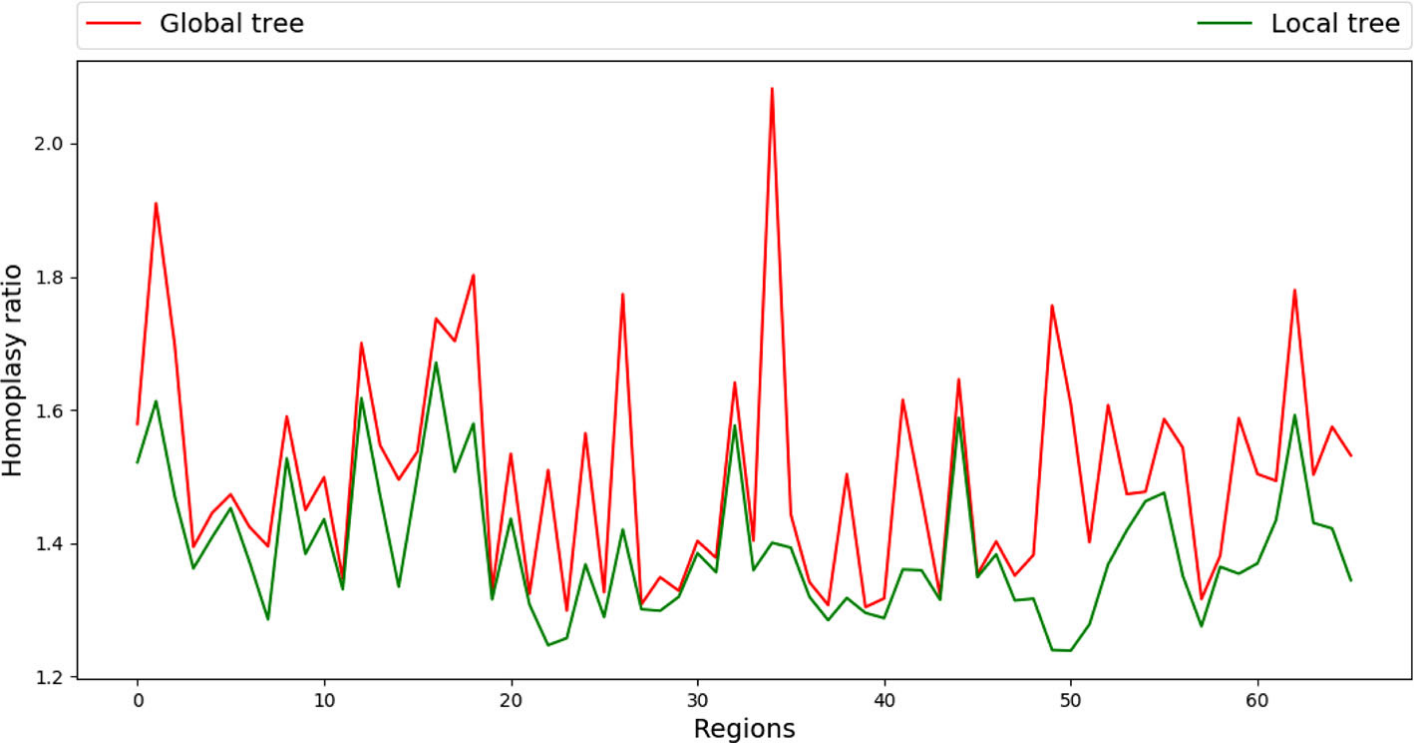
Homoplasy ratio based on global and regional trees for each region of *S. aureus*

**Fig. 12:**
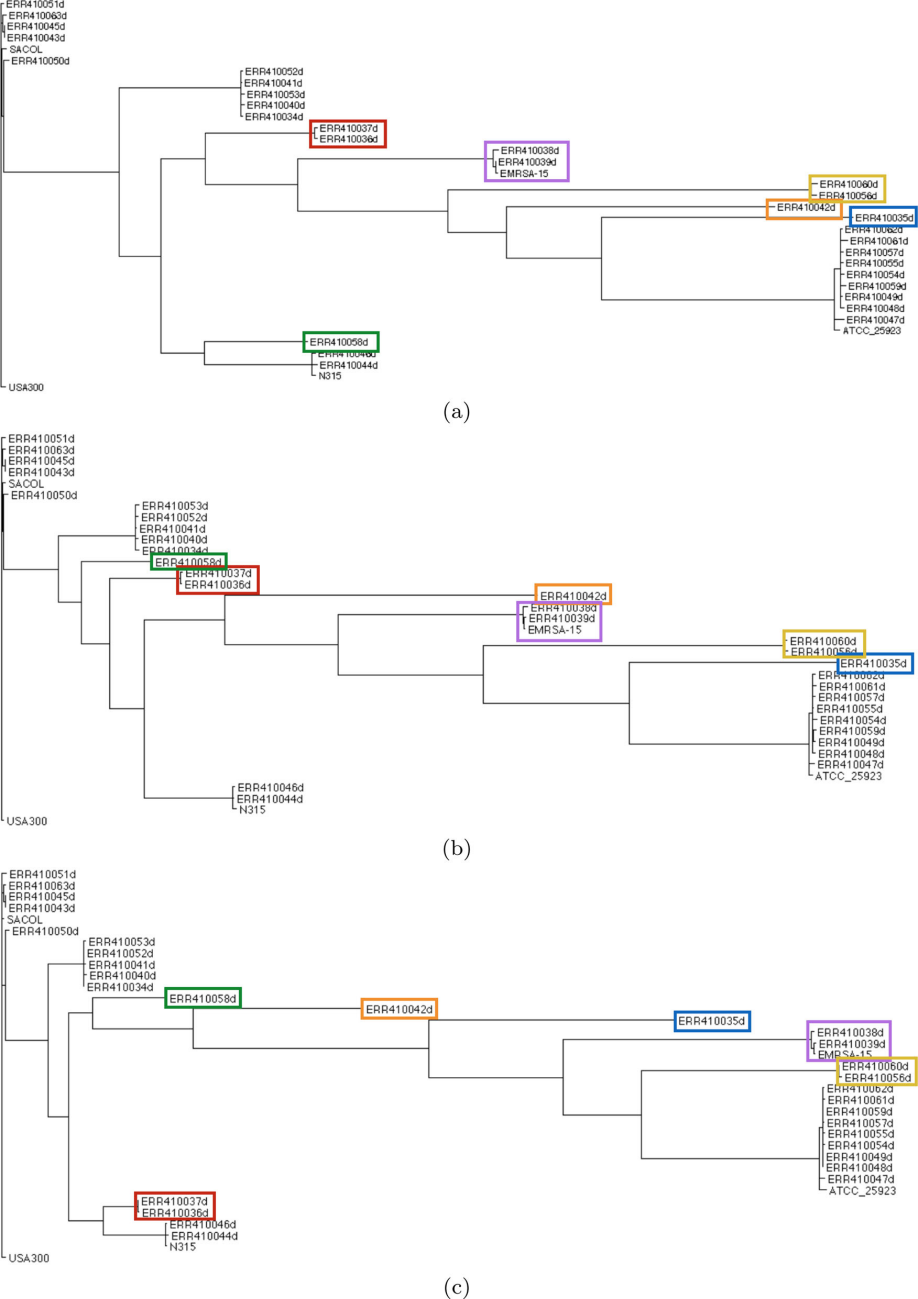
Phylogenetic trees in the 37^*th*^-39^*th*^ regions (**a**-**c**) of *S. aureus*

The plots of the homoplasy ratio and the excess changes for each region based on the global tree and a regional tree are shown in Fig. 11. For each region, both homoplasy ratio and excess changes decrease from the global tree to the regional tree, showing that the regions identified by ptACR have different topologies from the global tree, and each local tree is able to accommodate more sites within the corresponding region. Figure 12 shows local phylogenetic trees for three consecutive regions, starting from the 37^*th*^ segment, as an example for further analysis. The recombined groups of isolates are labeled in rectangles of the same color. According to the tree topologies, the 37^*th*^ region shows that the strain ERR410042 receives a copy from an ancestor of two strains, ERR410056 and ERR410060. Yet in the 38^*th*^ region the strain ERR410042 receives a copy from an ancestor of three strains, ERR410044, ERR410046 and N315, while a parent of ERR410056 and ERR410060 receives a copy from an ancestor of ERR410038, ERR410039 and EMRSA-15. In the 39^*th*^ region the strain ERR410042 receives the copies from parents of the strain ERR410058 instead. The information of region size, number of informative sites (SNPs), genes, overall compatibility ratio (Compat), the excess changes based on global tree (*EC*_*global*_) and local tree (*EC*_*local*_), and the reduction ratio of excess changes (Ratio) for the three regions is listed in Table 1. The number of excess changes decreases from the global tree to the local tree, showing that the local trees significantly reduce the apparent homoplasy based on the global tree.

**Table 1:** Information for regions of *S. aureus*

Region	Size (kb)^a^	SNPs^b^	Genes^c^	Compat^d^	*E* *C*_*global*_^e^	*E* *C*_*local*_^f^	Ratio^g^
37^*th*^	228.41	5526	*USA300_1420-1668*	94.59*%*	1993	1808	9.28%
38^*th*^	97.74	4777	*USA300_1669-1747*	93.63*%*	1512	1400	7.41%
39^*th*^	36.17	1745	*USA300_1747-1778*	89.93*%*	914	577	36.87%

^a^region size

^b^number of informative sites

^c^genes in the region

^d^regional compatibility ratio

^e^the excess changes based on the global tree

^f^the excess changes based on the local tree

^g^the reduction ratio of excess changes, 1-$\frac {{EC}_{local}}{{EC}_{global}}$

(paragraph on S. aureus moved to Discussion...)

**Fig. 13:**
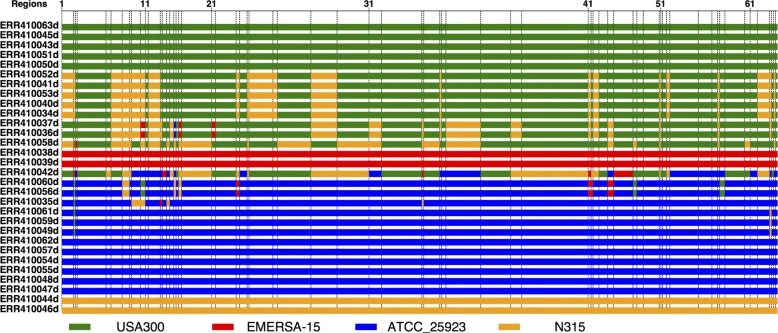
Mosaic patterns plotted from the most closely related reference strains across 66 regions for 30 *S. aureus* strains

To visualize the relationships among strains, a plot of the most closely related reference strain for each strain in each region is shown in Fig. 13. Strains ST8:USA300, EMRSA-15, ATCC 25923 and N315 were used as references, spanning several different lineages/strain types worldwide. For each strain, the most closely related reference strain is defined as the one that has the least differences in a region. Figure 13 shows that for several strains, the most closely related reference strain changes across the genome (i.e., pattern is mosaic), indicating that they are likely recombined (especially ERR410042). This is consistent with previous studies that found extensive recombination in this collection of *S. aureus* isolates [12, 13].

## Discussion

Our evaluation of ptACR demonstrates that it is not only sensitive to the true positives but robust to the false positive signals. Experiments with simulated data show that the sensitivity of ptACR increases for recombination between more divergent strains (with higher evolutionary distance). The performance is also influenced by substitution rate and heterogeneity. Including substitution rate heterogeneity among sites is more biologically realistic since some essential genes are more conserved while other genes tolerate more genetic changes during evolution over time. The extent of substitution rate heterogeneity is inversely related to the shape parameter in the gamma distribution. As the substitution rate heterogeneity decreases, more informative sites are obtained, and the ptACR performs better in terms of true positive rates. However, in the scenarios of higher substitution rate heterogeneity, there are more false positive signals. With the assessment of statistical significance of breakpoints using the permutation test, ptACR outperforms our previous method by filtering out more false positive signals. Also, substitution rate is varied to explore the influence of selection and genetic drift during evolution. When the substitution rate is lower, the true positive rate is higher and false positive rate is lower. As the substitution rate increases, more informative sites with a higher proportion of multi-state characters are obtained, and the alignment becomes more divergent. Furthermore, ptACR is robust even in the presence of coincident SNPs, where homoplasy is caused by the rapid evolution due to the high mutation rate instead of the structure of tree [38]. In our compatibility model, the coincident SNPs in the region would become background noise since all regional sites are shuffled to generate the null distribution of the statistic in the permutation test.

Bruen et al. [20] proposed a similar method called pairwise homoplasy index (PHI) based on pairwise incompatibility scores of the entire genome to detect the presence of recombination. They apply a permutation test on the entire alignment to obtain the Monte Carlo *p*-value for determining the significance of the observed PHI statistic. However, their method is designed only to detect whether recombination occurs anywhere in the genome. We extend the estimation from global to local scope to explore the recombination in local regions of the genome. That is, our method not only globally detects the presence of recombination events in an alignment, but locally identifies candidate breakpoints to obtain regions with distinct phylogenetic trees.

The ability to efficiently determine recombination breakpoints in bacterial genomes is especially important for analyses such as GWAS (genome-wide association studies) that attempt to statistically associate SNPs or loci with drug resistance or other phenotypes in a way informed by phylogenetic structure [39]. Uninformed of potential recombination, such studies run the risk drawing conclusions from the appearance of homoplasic sites in recombined regions (with respect to a single global phylogeny), which could be misinterpreted as evidence for positive selection at those sites.

We used ptACR to identify multiple genomic regions in a collection of *S. aureus* clinical isolates. Recombination has been previously reported for this species, but ptACR offers an efficient method to identify breakpoints where the recombination events are likely to have occurred. There were 65 such breakpoints in the set of 30 isolates we analyzed. The fact that phylogenetic trees generated from SNPs in adjacent regions are distinct, coupled with the mosaic pattern of similarities to reference strains among these regions, demonstrates the validity of the ptACR method. *S. aureus* is a human pathogen that causes lung and skin infections. Studies have revealed that *S. aureus* contains many types of mobile genetic elements that drive recombination hotspots, including plasmids, bacteriophages, pathogenicity genomic islands and islets, transposons, insertion sequences and staphylococcal cassette chromosomes (SCC) [12, 13]. In the collection we studied, the 28^*th*^ region contains *mecA* (*USA300HOU_0956*) gene that is located on SCC and most commonly known as encoding methicillin resistance in *S. aureus* [40, 41]. Also, the *scpA* gene, which is on a plasmid-associated island and contributes to staphylococcal virulence [42], is in the 37^*th*^ region.

The ptACR method has several limitations. One limitation is that, though the ability of ptACR is extended to handle an alignment consisting of multi-state characters, the pairwise compatibility for multi-state characters cannot guarantee setwise compatibility. Determining the compatibility of a pair of multi-state characters is solvable in polynomial time, however, determining the compatibility of a set of multi-state characters is NP-complete [24]. However, if two characters are incompatible, then there is no tree that can accommodate both sites at the same time. In practice, local decreases in average pairwise incompatibility is an approximate way to detect the boundaries in polynomial time.

Another limitation is that, because of the use of a sliding window, there is a practical limit on how small of a recombined region can be detected. The region has to be large enough, and the sequences diverse enough, so that region contains at least as many informative sites as the chosen window size. Finally, ptACR might be unable to give a proper interpretation in the case of overlapping recombination regions. The phylogenetic relationships in a region overlapped by two recombination events could look different from those in the non-intersecting parts of the regions. While ptACR would likely be able to detect the boundaries of the recombined regions, it would not necessarily be able to reconstruct the exact history of the events.

## Conclusions

The ptACR method is able to practically determine the compatibility of sites of binary- and multi-state characters and detect the recombination boundaries of lower average compatibility ratio with the assessment of statistical significance as candidate breakpoints. The method is sensitive, yet has a low false positive rate, supporting its ability to characterize mosaic genomes and identify the regions of distinct phylogenetic histories. With the detection of recombination events in clinical isolates of *S. aureus*, it could provide the better understanding of evolutionary relationships among bacterial isolates that is not clonal, driven by selection pressure or antibiotic resistance.

## Abbreviations

ACR: Average compatibility ratio
SNP: Single nucleotide polymorphism
